# Molecular and Functional Characterization of Kv1.3 Channels in Rat Articular Cartilage and Primary Chondrocytes: Regulation During Culture and Pro-Inflammatory Stimulation

**DOI:** 10.3390/ijms27219790

**Published:** 2026-10-31

**Authors:** Arturo Ponce, Lorena Hinojosa, Maria Luisa Roldán, Liora Shoshani

**Affiliations:** Department of Physiology, Biophysics and Neurosciences, Centro de Investigacion y Estudios Avanzados del IPN, Ciudad de México 07360, Mexico; lorena.hinojosa@cinvestav.mx (L.H.); luisa.roldan@cinvestav.mx (M.L.R.); shoshani@cinvestav.mx (L.S.)

**Keywords:** Kv1.3, articular chondrocytes, potassium channels, PAP-1, ShK-186, interleukin-1, inflammation, cartilage

## Abstract

We examined Kv1.3 expression and function in articular cartilage and primary chondrocytes from male Wistar rats using reverse transcription polymerase chain reaction (RT-PCR), Western blotting, immunofluorescence, and whole-cell patch-clamp recordings. Molecular assays supported Kcna3 transcript and Kv1.3 protein expression. PAP-1 and ShK-186 revealed blocker-sensitive outward currents, depolarized freshly isolated chondrocytes, and increased input resistance. During primary culture, total and PAP-1-sensitive conductance densities decreased, without a statistically significant change in the relative PAP-1-sensitive contribution. In unadjusted paired analyses, IL-1α and IL-1β increased PAP-1-sensitive conductance; IL-1β also reduced total conductance and increased the relative contribution. The relative increase with IL-1α was sensitive to the statistical approach. No statistically significant conductance changes were detected after LPS exposure. Blocker effects varied by cytokine and endpoint: PAP-1 reduced propidium iodide (PI) positivity following IL-1α exposure, whereas both blockers attenuated IL-1β-associated changes in metabolic activity, PI positivity, and cell density in paired analyses; the ShK-186 metabolic effect was sensitive to the statistical approach. These findings support a functional contribution of Kv1.3-sensitive conductance to chondrocyte electrical properties and responses to inflammatory stress.

## 1. Introduction

Articular cartilage is a highly specialized connective tissue that covers the surfaces of diarthrodial joints, providing a smooth, lubricated surface for articulation and facilitating the transmission and distribution of mechanical loads with minimal friction. Its functional properties depend on a highly organized extracellular matrix composed predominantly of water, type II collagen, and proteoglycans, particularly aggrecan. Articular chondrocytes are the principal resident cells of mature cartilage and are responsible for maintaining the balance between the synthesis and degradation of extracellular-matrix components. Because articular cartilage is avascular and has a limited intrinsic capacity for repair and regeneration, disruption of chondrocyte homeostasis can promote progressive extracellular-matrix deterioration and contribute to the development of degenerative joint diseases such as osteoarthritis [1,2,3].

Although chondrocytes are classically considered non-excitable cells, their plasma membrane contains a diverse repertoire of ion channels, pumps, exchangers, and transporters. These membrane proteins participate in the regulation of resting membrane potential, cell volume, intracellular Ca^2+^ signaling, mechanotransduction, extracellular-matrix synthesis, proliferation, differentiation, and cell survival [4,5,6]. Articular chondrocytes express members of multiple ion-channel families, including transient receptor potential channels, mechanosensitive PIEZO channels, chloride channels, two-pore-domain potassium channels, Ca^2+^-activated potassium channels, inwardly rectifying potassium channels, and voltage-gated potassium channels [6,7,8,9]. Alterations in the expression or activity of these channels can modify membrane potential, intracellular Ca^2+^ signaling, cell-volume regulation, and mechanotransduction and may therefore influence chondrocyte responses to mechanical, osmotic, metabolic, and inflammatory stimuli [7,9,10,11].

Voltage-gated potassium channels are important contributors to the electrical properties of articular chondrocytes. Voltage-dependent outward K^+^ currents have been described in cultured rabbit, equine, and elephant articular chondrocytes, as well as in acutely isolated canine and freshly dissociated rat articular chondrocytes [12,13,14,15]. In freshly dissociated rat chondrocytes, at least two kinetically distinct voltage-dependent K^+^ currents were identified. The predominant component activated at relatively negative membrane potentials, displayed time-dependent inactivation, and was inhibited by tetraethylammonium, 4-aminopyridine, α-dendrotoxin, charybdotoxin, and margatoxin [12]. These biophysical and pharmacological properties suggested the contribution of channels belonging to the Kv1 subfamily. However, the molecular identity of the channel responsible for this current was not established.

Kv1.3 is a voltage-gated K^+^ channel encoded by the *Kcna3* gene. The channel has been extensively characterized in lymphocytes, where it contributes to membrane-potential regulation and helps maintain the electrochemical driving force required for sustained Ca^2+^ entry. Through these mechanisms, Kv1.3 participates in lymphocyte activation, proliferation, cytokine production, and immune responses [16,17,18]. Kv1.3 expression, however, is not restricted to immune cells, and the channel has been identified in several excitable and non-excitable cell types. Depending on its cellular and subcellular localization, Kv1.3 has been implicated in cell-cycle progression, cellular metabolism, proliferation, apoptosis, and survival. Plasma-membrane Kv1.3 is particularly relevant to membrane-potential regulation and Ca^2+^-dependent signaling, whereas mitochondrial Kv1.3 has been associated with mitochondrial function and apoptotic signaling [19,20].

Evidence for Kv1.3 expression in cells of the chondrogenic lineage remains limited. Kv1.3 mRNA and protein have been detected in chondrogenic cells derived from embryonic chicken limb-bud mesenchymal cells. However, Kv1.3 protein was not clearly detected in the plasma-membrane fraction in that developmental model, emphasizing that total cellular expression does not necessarily demonstrate the presence of functional channels at the cell surface [21]. More recently, Kv1.6 was identified as a functionally relevant voltage-gated K^+^ channel in mouse articular chondrocytes. In that study, IL-1β reduced Kv1.6 expression and K^+^ current density, resulting in membrane depolarization, enhanced Ca^2+^ influx through CaV1.2 channels, mitochondrial dysfunction, and increased apoptosis [22]. Nevertheless, the molecular expression, tissue distribution, and functional contribution of Kv1.3 in mature articular cartilage and native articular chondrocytes remain insufficiently characterized.

Inflammatory mediators profoundly affect chondrocyte physiology and cartilage homeostasis. Cytokines such as interleukin-1β (IL-1β) and tumor necrosis factor-α (TNF-α) activate inflammatory and catabolic signaling pathways that promote extracellular matrix degradation, oxidative stress, metabolic alterations, mitochondrial dysfunction, and chondrocyte death [23,24,25,26]. Microbial products such as lipopolysaccharide (LPS) can similarly activate innate immune signaling in chondrocytes through Toll-like receptors, promoting inflammatory responses and cell death [27]. Ion channels may therefore function both as targets and as downstream effectors of inflammatory signaling.

Kv1.3 is particularly relevant in this context because its expression and activity are dynamically regulated during inflammatory responses in several cell types. In microglia and other myeloid cells, pro-inflammatory activation is associated with increased Kv1.3 expression and current density, whereas genetic deletion or pharmacological inhibition of Kv1.3 attenuates several pro-inflammatory cellular responses [28,29,30]. Ion-channel expression and function are also modified by inflammatory stimulation in articular chondrocytes. For example, inflammatory mediators enhance functional TRPA1 currents in rat articular chondrocytes, whereas IL-1β reduces Kv1.6-mediated currents in mouse articular chondrocytes [22,31]. These observations suggest that inflammatory conditions can remodel the ion-channel repertoire of chondrocytes and thereby alter their electrical, Ca^2+^-dependent, and survival responses. Whether pro-inflammatory mediators regulate functional Kv1.3 channels in articular chondrocytes, and whether Kv1.3 activity contributes to chondrocyte viability and survival under inflammatory stress, remains unknown.

The present study was therefore designed to characterize Kv1.3 expression and function in rat articular cartilage and articular chondrocytes. Rat articular cartilage and chondrocyte preparations were selected because our laboratory had previously worked with this experimental model [12,31]. Kcna3 transcript and Kv1.3 protein expression were examined using RT-PCR, Western blotting, and immunofluorescence analysis of articular cartilage. Whole-cell patch-clamp recordings were used to identify a pharmacologically defined current component attributed to Kv1.3 in freshly isolated chondrocytes and to evaluate changes in this component during primary culture. The contribution of Kv1.3 to the resting electrical properties of chondrocytes was also assessed by determining the effects of Kv1.3 blockers on membrane potential and input resistance. We subsequently investigated whether pro-inflammatory stimulation altered PAP-1-sensitive conductance and whether pharmacological blockade affected cellular metabolic activity, plasma-membrane integrity, and cell density under inflammatory conditions. We hypothesized that Kv1.3 is functionally expressed in native articular chondrocytes, contributes to their resting membrane properties, is dynamically regulated during primary culture and inflammatory stimulation, and participates in the cellular response of chondrocytes to pro-inflammatory stress.

## 2. Results

### 2.1. Kv1.3 Is Expressed in Rat Articular Cartilage

We first examined whether Kv1.3 is expressed in rat articular cartilage at both the transcript and protein levels. RT-PCR analysis was performed using two independent primer pairs designed against the rat *Kcna3*/Kv1.3 transcript (NM_019270.4). The primer sequences, nucleotide positions, and expected amplicon sizes are summarized in Table 1. The two primer sets were designed to generate products of 390 and 523 bp, respectively, providing independent molecular evidence for Kv1.3 transcript expression. RT-PCR amplification yielded products of the expected sizes in cartilage samples (Figure 1A), supporting the presence of *Kcna3* mRNA in rat articular cartilage.

Kv1.3 expression was subsequently examined at the protein level. Western blot analysis revealed a prominent immunoreactive band at approximately 70 kDa, consistent with the expected molecular mass of Kv1.3 (Figure 1B). In addition, immunofluorescence analysis of rat knee cartilage sections showed Kv1.3 immunoreactivity in green associated with articular chondrocytes, whose nuclei were counterstained in red with propidium iodide (Figure 1C). A higher-magnification image further illustrates the distribution of Kv1.3 immunoreactivity within individual chondrocytes (Figure 1D). Collectively, the RT-PCR, Western blot, and immunofluorescence results provide complementary evidence that Kv1.3 is expressed in rat articular cartilage at both the mRNA and protein levels.

### 2.2. Pharmacological Analysis Identifies a Kv1.3-Sensitive Current Component in Freshly Isolated Chondrocytes

We next examined whether Kv1.3 contributes to the voltage-dependent K^+^ current of freshly isolated rat articular chondrocytes. Two structurally distinct Kv1.3 blockers were used: PAP-1, a small-molecule inhibitor reported to block Kv1.3 with an EC_50_ of approximately 2 nM, and ShK-186, a peptide inhibitor with a reported IC_50_ of approximately 69 pM [32,33]. These potency values were obtained in previous studies and do not represent concentration-response measurements in chondrocytes.

Whole-cell currents were elicited from a holding potential of −80 mV using voltage steps from −80 to +80 mV in 10-mV increments. Under control conditions, freshly isolated chondrocytes displayed outward currents that progressively increased with membrane depolarization. Application of ShK-186 (150 pM) reduced the outward current, and digital subtraction of the current recorded in the presence of ShK-186 from the corresponding control current revealed a voltage-dependent ShK-186-sensitive component (Figure 2A1). PAP-1 (5 nM) produced a comparable reduction in outward current, yielding a PAP-1-sensitive component using the same subtraction procedure (Figure 2B1). Based on their sensitivity to two structurally distinct Kv1.3 blockers, together with the molecular evidence for Kv1.3 expression described above, these blocker-sensitive components were operationally attributed to Kv1.3 and are hereafter referred to as I_Kv1.3_.

Current density–voltage relationships showed the total outward current under control conditions, the residual current in the presence of each blocker, and the corresponding blocker-sensitive component obtained by subtraction (Figure 2A2,B2). Both ShK-186 and PAP-1 reduced current density over the range of depolarizing potentials, whereas the blocker-sensitive current increased progressively with depolarization. Conversion of peak current density to conductance density revealed a voltage-dependent increase followed by saturation at positive potentials for the total, residual, and blocker-sensitive components (Figure 2A3,B3).

To compare voltage dependence independently of current magnitude, conductance values for each component were normalized to their respective maximal conductance (g/g_max_). The resulting activation relationships were fitted with Boltzmann functions (Figure 2A4,B4). The blocker-sensitive components activated over a voltage range comparable to that of the total outward current. Taken together, the detection of voltage-dependent outward-current components sensitive to both ShK-186 and PAP-1, together with the molecular evidence for Kv1.3 expression, supports a functional contribution of Kv1.3 to the K^+^ conductance of freshly isolated rat articular chondrocytes.

### 2.3. Kv1.3 Blockade Depolarizes the Membrane and Increases Input Resistance in Freshly Isolated Chondrocytes

To determine whether Kv1.3 contributes to the resting electrical properties of freshly isolated rat articular chondrocytes, membrane potential (Vm) and input resistance (Rin) were monitored under whole-cell current-clamp conditions during exposure to the structurally distinct Kv1.3 blockers ShK-186 and PAP-1. Repeated hyperpolarizing current pulses of constant amplitude were applied to monitor Rin while simultaneously recording Vm. Values are expressed as mean ± SEM calculated from five rat means, with five cells recorded per rat for each blocker. The 95% confidence intervals (CIs) reported below refer to mean within-rat blocker-induced changes.

ShK-186 (150 pM) depolarized the membrane, and this effect was largely reversed after washout in the representative recording (Figure 3A1). Mean Vm changed from −43.17 ± 0.81 mV under control conditions to −41.14 ± 0.69 mV during ShK-186 application, corresponding to a depolarization of 2.03 ± 0.16 mV (95% CI: 1.590–2.473 mV; Figure 3A2). Rin increased from 8.15 ± 0.25 to 9.57 ± 0.23 GΩ, corresponding to an increase of 1.42 ± 0.05 GΩ (95% CI: 1.290–1.550 GΩ; Figure 3A3).

PAP-1 (5 nM) also depolarized the membrane, with substantial recovery after washout in the representative recording (Figure 3B1). Mean Vm shifted from −43.07 ± 0.67 to −39.27 ± 0.69 mV, yielding a depolarization of 3.80 ± 0.18 mV (95% CI: 3.315–4.290 mV; Figure 3B2). Rin increased from 7.94 ± 0.10 to 9.81 ± 0.08 GΩ, corresponding to an increase of 1.88 ± 0.14 GΩ (95% CI: 1.500–2.252 GΩ; Figure 3B3). For both blockers, the increase in Rin was also evident as larger voltage deflections in response to identical hyperpolarizing current pulses.

All blocker-induced changes in Vm and Rin differed significantly from zero (*p* < 0.001; two-tailed one-sample Student’s *t*-tests applied to the five rat-mean changes). The membrane depolarization and accompanying increase in Rin are consistent with inhibition of a K^+^ conductance contributing to the resting electrical properties of these cells.

Together, the effects of ShK-186 and PAP-1 support a functional contribution of Kv1.3-sensitive conductance near the resting membrane potential.

### 2.4. Total and PAP-1-Sensitive Conductance Densities Decrease During Primary Chondrocyte Culture

To examine culture-dependent changes in K^+^ conductance density, four independent primary chondrocyte preparations were generated. Each preparation was obtained by pooling chondrocytes isolated from the hip articular cartilage of three rats. Cells were counted and seeded onto glass coverslips at a density of 500 cells/mm^2^. The coverslips were maintained in 35-mm Petri dishes under standard culture conditions.

Separate coverslips from each preparation were used for electrophysiological recordings on days 0, 1, 3, and 5 after plating. At each time point, five different cells were analyzed per preparation, yielding 20 cells per day. Whole-cell K^+^ currents were recorded using the voltage-clamp protocol described above, under control conditions and during application of PAP-1 (5 nM). Each preparation constituted one independent biological replicate (n = 4). Cell-level measurements were averaged within each preparation and time point, and the resulting preparation-level means were used for statistical analysis, with preparation as the matched unit across time points. Values are presented as mean ± SEM across the four preparation-level means. Time effects were assessed using one-way repeated-measures ANOVA with Greenhouse–Geisser correction. For the two conductance-density outcomes, comparisons of days 1, 3, and 5 with day 0 were performed using two-tailed paired Student’s *t*-tests, with Holm adjustment across the three comparisons for each outcome.

Representative current-density families are shown in Figure 4A. Total outward K^+^ currents remained detectable throughout the 5-day culture period. The PAP-1-sensitive component, obtained by subtracting currents recorded during PAP-1 application from the corresponding control currents and operationally attributed to Kv1.3, was also detected at all time points.

Mean membrane capacitance was 30.96 ± 0.59 pF on day 0, 32.72 ± 0.42 pF on day 1, 32.70 ± 0.56 pF on day 3, and 30.67 ± 0.59 pF on day 5 (Figure 4B). No statistically significant effect of culture time on membrane capacitance was detected (F(1.929, 5.786) = 3.231, *p* = 0.115).

Total maximal K^+^ conductance density decreased during primary culture (Figure 4C). Mean values were 229.12 ± 2.35 pS/pF on day 0, 214.38 ± 3.94 pS/pF on day 1, 207.88 ± 2.15 pS/pF on day 3, and 202.95 ± 1.61 pS/pF on day 5. The ANOVA revealed a significant effect of culture time (F(1.593, 4.779) = 18.664, *p* = 0.0063). Mean paired changes relative to day 0 were −14.74 pS/pF on day 1 (95% CI: −32.26 to 2.77 pS/pF; Holm-adjusted *p* = 0.0751), −21.23 pS/pF on day 3 (95% CI: −35.30 to −7.17 pS/pF; Holm-adjusted *p* = 0.0343), and −26.17 pS/pF on day 5 (95% CI: −37.57 to −14.77 pS/pF; Holm-adjusted *p* = 0.0159).

Mean PAP-1-sensitive maximal conductance density was 51.61 ± 1.21 pS/pF on day 0, 46.05 ± 1.27 pS/pF on day 1, 47.90 ± 1.26 pS/pF on day 3, and 41.74 ± 0.70 pS/pF on day 5 (Figure 4D). The ANOVA revealed a significant effect of culture time (F(1.550, 4.649) = 12.894, *p* = 0.0143). Mean paired changes relative to day 0 were −5.56 pS/pF on day 1 (95% CI: −6.49 to −4.64 pS/pF; Holm-adjusted *p* = 0.000926), −3.71 pS/pF on day 3 (95% CI: −9.67 to 2.24 pS/pF; Holm-adjusted *p* = 0.1412), and −9.88 pS/pF on day 5 (95% CI: −15.94 to −3.82 pS/pF; Holm-adjusted *p* = 0.0278).

The relative contribution of the PAP-1-sensitive component was calculated for each cell as 100 × PAP-1-sensitive maximal conductance density divided by total maximal conductance density, before averaging within each preparation and time point (Figure 4E). Its mean contribution was 22.63 ± 0.70% on day 0, 21.56 ± 0.66% on day 1, 23.09 ± 0.52% on day 3, and 20.64 ± 0.35% on day 5. No statistically significant effect of culture time on this relative contribution was detected (F(1.551, 4.654) = 3.076, *p* = 0.143).

Primary culture was associated with an overall reduction in total and PAP-1-sensitive maximal conductance densities, with statistically supported reductions at different time points. No statistically significant overall time effect was detected for the relative PAP-1-sensitive contribution to total maximal K^+^ conductance density.

### 2.5. IL-1α and IL-1β Increase PAP-1-Sensitive Conductance in Primary Chondrocytes

To determine whether pro-inflammatory stimulation modifies total K^+^ conductance and the contribution of Kv1.3 channels, primary rat articular chondrocytes were maintained in culture for 24 h and subsequently exposed for 12 h to IL-1α (10 ng/mL), IL-1β (10 ng/mL), or LPS (1 µg/mL). Whole-cell K^+^ currents were recorded before and during application of PAP-1 (5 nM). The PAP-1-sensitive component was obtained by subtracting currents recorded during PAP-1 application from the corresponding currents recorded before blocker application and was operationally attributed to Kv1.3.

Four independent biological replicates were performed for each inflammatory agent, each using chondrocytes isolated from the hip articular cartilage of a different rat. Separate sets of four rats were used for the three agents, yielding 12 rats in total. Within each preparation, cells were seeded onto glass coverslips and distributed between matched untreated and inflammatory-treatment conditions. Five cells were recorded per preparation and condition, yielding 20 cell recordings per condition for each agent. Measurements were averaged within each preparation and condition to obtain one value per rat, and each rat constituted one independent biological replicate (n = 4 per agent).

Values are expressed as mean ± SEM across the four biological replicates. Treatment comparisons were evaluated using two-tailed paired Student’s *t*-tests applied to the four matched pairs of rat-level means. The signs of the reported t statistics follow the control-minus-treatment convention. For reporting effect estimates, mean paired changes and their 95% confidence intervals (CIs) are expressed as treatment minus control. An exact two-sided Wilcoxon signed-rank test was additionally performed as a sensitivity analysis for the relative PAP-1-sensitive contribution following IL-1α exposure, because the paired differences failed the Shapiro–Wilk normality test. All *p* values and confidence intervals reported below are unadjusted for multiple comparisons.

Following IL-1α exposure, mean maximal total K^+^ conductance density decreased from 210.307 ± 2.005 to 202.834 ± 1.928 pS/pF, corresponding to a mean paired change of −7.473 pS/pF (95% CI: −19.563 to 4.618 pS/pF), but the difference was not statistically significant (t(3) = 1.967, *p* = 0.144; Figure 5A). IL-1β significantly reduced maximal total K^+^ conductance density from 211.107 ± 1.780 to 199.701 ± 3.580 pS/pF, with a mean paired change of −11.406 pS/pF (95% CI: −19.623 to −3.188 pS/pF; t(3) = 4.417, *p* = 0.0215). Following LPS exposure, mean conductance density decreased from 212.575 ± 2.680 to 201.142 ± 2.007 pS/pF, with a mean paired change of −11.433 pS/pF (95% CI: −24.188 to 1.322 pS/pF), although the difference was not statistically significant (t(3) = 2.853, *p* = 0.0650).

The PAP-1-sensitive conductance attributed to Kv1.3 increased following stimulation with either IL-1α or IL-1β (Figure 5B). IL-1α increased maximal PAP-1-sensitive conductance density from 41.813 ± 0.474 to 48.602 ± 0.943 pS/pF, corresponding to a mean paired increase of 6.788 pS/pF (95% CI: 3.379–10.197 pS/pF; t(3) = −6.337, *p* = 0.00795). IL-1β increased it from 41.657 ± 0.521 to 49.231 ± 1.619 pS/pF, corresponding to a mean paired increase of 7.574 pS/pF (95% CI: 3.711–11.437 pS/pF; t(3) = −6.240, *p* = 0.00830). These changes corresponded to increases of approximately 16% and 18%, respectively. Following LPS exposure, mean PAP-1-sensitive conductance density decreased from 42.953 ± 0.670 to 40.416 ± 1.115 pS/pF, with a mean paired change of −2.536 pS/pF (95% CI: −5.300 to 0.228 pS/pF), but the difference was not statistically significant (t(3) = 2.920, *p* = 0.0615).

The PAP-1-sensitive component was also expressed as a percentage of total maximal K^+^ conductance (Figure 5C). Following IL-1α exposure, the mean contribution increased from 19.878 ± 0.174% to 23.960 ± 0.408%, corresponding to a mean paired increase of 4.082 percentage points (t-based 95% CI: 2.615–5.548 percentage points). Although the paired *t*-test yielded *p* = 0.00303 (t(3) = −8.858), the paired differences showed a departure from normality (Shapiro–Wilk W = 0.726, *p* = 0.0223). The exact two-sided Wilcoxon sensitivity analysis yielded *p* = 0.125. Thus, the increase observed in all four biological replicates was interpreted cautiously because its inferential support depended on the statistical approach.

IL-1β increased the relative PAP-1-sensitive contribution from 19.728 ± 0.130% to 24.644 ± 0.540%, corresponding to a mean paired increase of 4.916 percentage points (95% CI: 3.588–6.244 percentage points; t(3) = −11.780, *p* = 0.00131). Following LPS exposure, the relative contribution changed from 20.222 ± 0.257% to 20.116 ± 0.383%, with a mean paired change of −0.106 percentage points (95% CI: −0.675 to 0.463 percentage points) and no statistically significant difference detected (t(3) = 0.593, *p* = 0.595).

Together, the unadjusted analyses support an increase in PAP-1-sensitive conductance following IL-1α and IL-1β exposure. For IL-1β, this increase was accompanied by a reduction in total conductance and an increased relative PAP-1-sensitive contribution. The increase in relative contribution observed with IL-1α was sensitive to the statistical approach and requires cautious interpretation. No statistically significant change was detected following LPS exposure in any of the three outcomes.

### 2.6. Kv1.3 Blockade Attenuates Pro-Inflammatory Cytokine-Induced Chondrocyte Damage

We examined whether PAP-1 or ShK-186 modified cytokine-associated changes in cellular metabolic activity, plasma-membrane integrity, and cell density. Four independent biological preparations were analyzed, each obtained by pooling chondrocytes from three rats. Five technical replicate wells per condition were averaged within each preparation. Values are expressed as mean ± SEM across the four preparation-level means. Global effects were assessed using two-way repeated-measures ANOVA with Greenhouse–Geisser correction. Specific comparisons used two-tailed paired *t*-tests with Holm–Šidák adjustment within families of three comparisons. Mean paired changes were calculated as the comparison condition minus its reference condition. Changes and their 95% confidence intervals (CIs) are expressed in percentage points for normalized MTT absorbance and PI positivity, and in nuclei/mm^2^ for cell density. Confidence intervals were not adjusted for multiple comparisons; statistical significance was assessed using adjusted *p* values.

For cellular metabolic activity, the interaction between inflammatory treatment and blockade did not reach statistical significance after Greenhouse–Geisser correction (F(1.593, 4.780) = 6.072, *p* = 0.0521; Figure 6A). IL-1α and IL-1β reduced normalized MTT absorbance from 100.63 ± 1.44% in control cultures to 75.10 ± 1.59% and 71.37 ± 1.22%, respectively. Mean paired changes relative to control were −25.53 (95% CI: −33.73 to −17.33; adjusted *p* = 0.00437) for IL-1α and −29.25 (95% CI: −36.81 to −21.69; adjusted *p* = 0.00346) for IL-1β.

Under IL-1α stimulation, mean MTT absorbance increased to 83.73 ± 1.80% with ShK-186 and 87.64 ± 1.80% with PAP-1. Mean paired changes relative to IL-1α alone were 8.64 (95% CI: 1.91–15.36) and 12.54 (95% CI: 3.79–21.29), respectively, but neither comparison reached statistical significance after adjustment (both adjusted *p* = 0.0581). Under IL-1β stimulation, ShK-186 and PAP-1 increased MTT absorbance to 79.78 ± 1.50% and 86.60 ± 1.52%, respectively. Mean paired changes relative to IL-1β alone were 8.41 with ShK-186 (95% CI: 2.80–14.02; adjusted *p* = 0.0256) and 15.23 with PAP-1 (95% CI: 12.58–17.88; adjusted *p* = 0.00107). PAP-1 produced a greater increase than ShK-186 under IL-1β stimulation (mean paired difference: 6.82; 95% CI: 2.75–10.89; adjusted *p* = 0.0256). The IL-1β versus IL-1β + ShK-186 comparison requires caution because its paired differences departed from normality (Shapiro–Wilk *p* = 0.0480), and an unadjusted exact Wilcoxon sensitivity analysis yielded *p* = 0.125. Neither blocker significantly changed MTT absorbance under basal conditions; mean paired changes relative to control were 2.86 with ShK-186 (95% CI: −4.62 to 10.33) and −0.12 with PAP-1 (95% CI: −6.28 to 6.03).

For PI positivity, a significant interaction between inflammatory treatment and blockade was detected (F(1.968, 5.904) = 16.835, *p* = 0.00369; Figure 6B). IL-1α and IL-1β increased the percentage of PI-positive nuclei from 5.86 ± 0.50% in control cultures to 24.80 ± 1.00% and 22.10 ± 0.75%, respectively. Mean paired changes relative to control were 18.94 for IL-1α (95% CI: 16.14–21.74) and 16.24 for IL-1β (95% CI: 14.26–18.22; both adjusted *p* < 0.001). The complementary proportion of PI-negative nuclei in untreated cultures was 94.14 ± 0.50% (mean ± SEM across four biological preparations), calculated as 100% minus PI positivity. This endpoint reflects retained plasma-membrane integrity among imaged cells at the assay endpoint; it is not an independent viability assay or a measure of viability immediately after isolation.

Under IL-1α stimulation, PI positivity was 19.00 ± 1.00% with ShK-186 and 16.30 ± 0.90% with PAP-1. Mean paired changes relative to IL-1α alone were −5.80 with ShK-186 (95% CI: −11.96 to 0.36; adjusted *p* = 0.0578) and −8.50 with PAP-1 (95% CI: −14.17 to −2.83; adjusted *p* = 0.0346). Under IL-1β stimulation, both blockers reduced PI positivity to 13.90 ± 0.80% with ShK-186 and 13.30 ± 0.85% with PAP-1. Corresponding mean paired changes relative to IL-1β alone were −8.20 (95% CI: −12.47 to −3.93; adjusted *p* = 0.0175) and −8.80 (95% CI: −11.99 to −5.61; adjusted *p* = 0.00931), respectively. The blockers did not differ significantly under IL-1β stimulation (PAP-1 minus ShK-186: −0.60; 95% CI: −5.64 to 4.44). Under IL-1α stimulation, PAP-1 yielded lower PI positivity than ShK-186 in the paired *t*-test analysis (mean paired difference: −2.70; t-based 95% CI: −3.56 to −1.84; adjusted *p* = 0.00629), although this comparison requires caution because the paired differences departed from normality and an unadjusted exact Wilcoxon sensitivity analysis yielded *p* = 0.125. Neither blocker significantly changed basal PI positivity; mean paired changes relative to control were 0.65 with ShK-186 (95% CI: −2.37 to 3.68) and 0.95 with PAP-1 (95% CI: −1.27 to 3.17).

For total cell density, the interaction did not reach statistical significance after Greenhouse–Geisser correction (F(2.022, 6.067) = 3.972, *p* = 0.0787; Figure 6C). IL-1α and IL-1β reduced cell density from 250 ± 7.5 nuclei/mm^2^ in control cultures to 155 ± 6 and 140 ± 6 nuclei/mm^2^, respectively. Mean paired changes relative to control were −95 for IL-1α (95% CI: −126.38 to −63.62) and −110 for IL-1β (95% CI: −142.09 to −77.91; both adjusted *p* = 0.00330).

Under IL-1α stimulation, mean cell density was 170 ± 6.5 nuclei/mm^2^ with ShK-186 and 189 ± 7 nuclei/mm^2^ with PAP-1. Mean paired changes relative to IL-1α alone were 15 with ShK-186 (95% CI: −21.03 to 51.03; adjusted *p* = 0.277) and 34 with PAP-1 (95% CI: −6.94 to 74.94; adjusted *p* = 0.149); neither comparison was statistically significant. Under IL-1β stimulation, both blockers attenuated the reduction in cell density, with values of 180 ± 5.5 nuclei/mm^2^ for ShK-186 and 183 ± 7 nuclei/mm^2^ for PAP-1. Mean paired changes relative to IL-1β alone were 40 with ShK-186 (95% CI: 19.32–60.68) and 43 with PAP-1 (95% CI: 23.15–62.85; both adjusted *p* = 0.0186). Neither blocker significantly changed cell density under basal conditions; mean paired changes relative to control were −5 with ShK-186 (95% CI: −44.31 to 34.31) and −3 with PAP-1 (95% CI: −34.87 to 28.87).

These results identify endpoint-specific effects of Kv1.3 blockers during cytokine exposure. Under IL-1α stimulation, PAP-1 significantly reduced PI positivity, whereas the comparisons for MTT absorbance and cell density did not reach statistical significance. Under IL-1β stimulation, both blockers attenuated changes in the three measured endpoints in the paired analyses, although the MTT comparison involving ShK-186 was sensitive to the statistical approach. These measurements do not establish the mechanism or mode of cell death. For IL-1α with ShK-186, the 8.64-percentage-point increase in normalized MTT absorbance was not statistically significant after adjustment (*p* = 0.0581) and should not be interpreted as demonstrated protection. MTT reduction is a culture-level metabolic endpoint, not a direct percentage of viable cells, and assay-specific analytical precision was not quantified in this study.

## 3. Discussion

The present study provides complementary molecular, histological, electrophysiological, and pharmacological evidence supporting the expression of functional Kv1.3 channels in rat articular chondrocytes. PAP-1- and ShK-186-sensitive outward currents were detected in freshly isolated cells, and blockade depolarized the membrane and increased input resistance. Primary culture was associated with reductions in total and PAP-1-sensitive conductance densities. Inflammatory stimulation also modified the PAP-1-sensitive component, although the findings require distinctions between changes in conductance density, relative contribution, and total conductance. Pharmacological blockade additionally attenuated several measures of cytokine-associated cellular injury.

Previous studies have established that articular chondrocytes express voltage-gated, Ca^2+^-activated, inward-rectifier, and two-pore-domain K^+^ channels [5,6,8]. Voltage-dependent outward K^+^ currents have been described in rabbit, canine, equine, elephant, mouse, and rat articular chondrocytes [12,13,14,15,34]. In freshly dissociated rat chondrocytes, we previously identified two kinetically distinct voltage-dependent currents, with the predominant component displaying pharmacological properties suggestive of Kv1-family channels, although its molecular identity remained unresolved [12]. The present detection of *Kcna3* transcripts, Kv1.3 protein, chondrocyte-associated immunoreactivity, and blocker-sensitive currents extends those observations.

This combination of evidence is relevant because total cellular expression does not establish functional channel expression at the plasma membrane. Varga et al. [21] detected Kv1.3 during chondrogenesis of embryonic chicken limb-bud mesenchymal cells but did not clearly demonstrate its presence in the plasma-membrane fraction. Their findings linked developmental changes in Kv-channel expression to membrane potential, Ca^2+^ oscillations, proliferation, and cartilage formation. The present study extends the investigation of Kv1.3 to rat articular chondrocytes by identifying a functional, pharmacologically defined component of their outward current.

The use of two structurally distinct inhibitors strengthens the functional interpretation. PAP-1 and ShK-186 reduced outward currents and revealed voltage-dependent blocker-sensitive components [32,33]. Their effects, together with the molecular findings, support a contribution of Kv1.3. Nevertheless, pharmacological subtraction identifies the fraction inhibited under the recording conditions and does not establish that this fraction represents the entire Kv1.3 conductance or excludes contributions from other drug-sensitive channels. The PAP-1-sensitive component should therefore retain its operational designation, with genetic suppression of Kcna3 providing an important future validation.

Both blockers produced approximately 2–4 mV of membrane depolarization accompanied by increased input resistance. This combination is consistent with inhibition of a K^+^ conductance contributing near the resting membrane potential. Similar contributions of voltage-dependent K^+^ currents have been reported in canine and mouse articular chondrocytes [14,34], whereas voltage-independent K2P conductance has a prominent role in isolated human chondrocytes [35]. These observations support a multichannel basis for chondrocyte membrane potential whose composition can vary with species and experimental conditions. Even modest voltage changes may influence the driving force for ion movement and its coupling to Ca^2+^ signaling and other cellular processes [5,8].

Primary culture altered the electrophysiological phenotype. Total and PAP-1-sensitive conductance densities were lower than their day-0 values at subsequent time points. The PAP-1-sensitive component did not decline monotonically because its mean increased between days 1 and 3 before decreasing on day 5. Its relative contribution ranged from approximately 21% to 23%, with no statistically significant effect of culture time detected. This result does not establish equivalence across time points or demonstrate that Kv1.3 is selectively preserved. Membrane capacitance also showed no statistically significant overall time effect, but this alone does not exclude changes in cell size or membrane organization. Together, the findings indicate that culture conditions should be considered when comparing chondrocyte conductances and interpreting channel regulation. The developmental channel changes reported by Varga et al. [21] provide additional context for such phenotypic plasticity. Kcna3 transcript abundance, total Kv1.3 protein abundance, and surface localization were not quantified across culture days. The conductance changes therefore cannot be interpreted as evidence of reduced Kv1.3 molecular expression; altered channel abundance, trafficking, or gating remain untested explanations.

The inflammatory experiments identified increased PAP-1-sensitive conductance following IL-1α and IL-1β exposure in the unadjusted paired analyses. The increases were approximately 16% and 18%, respectively. For IL-1β, the increase was accompanied by reduced total conductance and a greater relative PAP-1-sensitive contribution. For IL-1α, no statistically significant reduction in total conductance was detected. These findings support an increase in the pharmacologically defined component following cytokine exposure, while the evidence for changes in its proportional contribution requires separate consideration.

In particular, the relative PAP-1-sensitive contribution increased in all four IL-1α biological replicates, but the paired differences departed from normality. The paired *t*-test yielded *p* = 0.00303, whereas the exact Wilcoxon sensitivity analysis yielded *p* = 0.125. The consistent direction of the observations provides descriptive evidence of an increase, but the inferential support depends on the statistical approach. The exact test also has limited resolution with four pairs. This comparison therefore requires cautious interpretation. More generally, the inflammatory experiments used four independent rats per agent, and the reported comparisons were not adjusted for multiplicity. Independent confirmation is needed to assess the reproducibility and precision of these findings.

No statistically significant change in total conductance, PAP-1-sensitive conductance, or relative contribution was detected following LPS exposure at the concentration and duration tested. These results do not establish the absence of an LPS effect or demonstrate that responses to LPS differ from those to the interleukins. Such conclusions require direct comparisons of treatment effects. Studies in microglia have reported increased Kv1.3 expression and activity during pro-inflammatory activation [28,30], illustrating the importance of cellular context. Whether receptor-dependent signaling accounts for the observations in chondrocytes remains unresolved. Although LPS can activate macrophages, its actions are not restricted to those cells: direct TLR4-dependent effects on chondrocyte matrix synthesis have been reported [36]. We did not measure TLR4 activation or inflammatory signaling after LPS exposure, so the absence of statistically significant conductance changes cannot establish whether LPS elicited an inflammatory response in these cultures.

The IL-1β findings can also be considered alongside the recent report of reduced Kv1.6-mediated currents in mouse articular chondrocytes. Kurata et al. [22] linked Kv1.6 downregulation to depolarization, enhanced Ca^2+^ influx through CaV1.2 channels, mitochondrial dysfunction, and apoptosis. The increased PAP-1-sensitive component observed here suggests that different channel populations may respond differently to inflammatory exposure. However, the studies used different species and experimental conditions, and Kv1.6 was not measured in the present experiments. A coordinated redistribution of Kv1 conductances remains a hypothesis requiring direct investigation. Compensatory or reciprocal regulation of Kv1.3 and Kv1.6 is one possible explanation, but neither such regulation nor a causal relationship between these channel populations was tested here.

Evidence from other joint compartments further motivates this work. Wu et al. [37] reported increased Kv1.3 expression in pro-inflammatory macrophages and osteoarthritic synovium and showed that Kv1.3 blockade attenuated macrophage polarization, synovitis, and cartilage degeneration in experimental osteoarthritis. The present observations support investigating articular chondrocytes as another potential cellular compartment involved in Kv1.3-associated responses. They do not establish therapeutic efficacy in osteoarthritic cartilage. The gap between these in vitro observations and treatment of osteoarthritis in vivo remains substantial; disease models, tissue-level outcomes, and assessment of safety and exposure would be needed before therapeutic conclusions could be drawn.

The mechanisms linking Kv1.3-sensitive conductance to these responses remain open. Changes in membrane potential could influence Ca^2+^ entry, as established in lymphocytes [16,17] and suggested by work on chondrocyte K^+^ conductances [22]. Kv1.3 has also been linked to proliferative signaling, mitochondrial respiration, and apoptosis in other cell types [38,39,40]. Intracellular Ca^2+^, mitochondrial function, reactive oxygen species, and apoptotic pathways were not measured here, so these mechanisms remain hypotheses. Likewise, the cytokine-associated increase in PAP-1-sensitive conductance cannot be assigned to transcription, protein abundance, membrane trafficking, or gating from the present data.

Because treatment allocation was not formally randomized and investigators were aware of treatment identity, potential selection and assessment biases cannot be excluded. RT-PCR, Western blotting, and immunofluorescence each used tissue from a single, different rat. These findings provide complementary descriptive evidence, although the absence of independent biological replication within each assay limits assessment of between-animal variability.

The present findings support a functional contribution of Kv1.3-sensitive conductance to chondrocyte electrical properties and show that this component changes during primary culture and IL-1 exposure. The effects of PAP-1 and ShK-186 on metabolic activity, membrane integrity, and cell density varied with the treatment combination and further suggest an involvement of Kv1.3-sensitive mechanisms in chondrocyte responses to inflammatory stress.

## 4. Materials and Methods

### 4.1. Animals and Tissue Collection

Articular cartilage and primary articular chondrocytes were obtained from the hip or knee joints of 6–8-week-old male Wistar rats weighing 120–160 g. A total of 46 rats were used throughout the study. All animals originated from the institutional breeding colony of CINVESTAV (Mexico City, Mexico), where they were maintained under conventional housing conditions with periodic health monitoring. Animals were housed in groups of five per cage at 22 ± 2 °C, with controlled relative humidity (40–60%) and a 12-h light/12-h dark cycle. Cages contained conventional bedding without additional environmental enrichment. Food and water were provided ad libitum. Animals were used directly from the institutional breeding colony without an additional acclimatization period and had not undergone previous experimental treatments or procedures.

Animal health and welfare were monitored daily by institutional animal facility personnel for signs of illness, physical injury, or abnormal behavior. Predefined humane endpoints included substantial body-weight loss, difficulty feeding, and immobility. The weight-loss criterion was qualitative, with no predefined numerical threshold. Animals meeting these criteria would have been removed from the study and humanely euthanized. No unexpected deaths, illnesses, injuries, or other adverse events were observed, and no animals reached the predefined humane endpoints.

To minimize animal use, tissues from individual animals were used for multiple experimental procedures when appropriate. Ten rats were used for the initial electrophysiological characterization and measurements of resting membrane properties, with separate sets of five rats used for ShK-186 and PAP-1. Within each set, four rats contributed to voltage-clamp experiments, and all five contributed to current-clamp measurements. Separate groups of 12 rats each were used for culture-dependent electrophysiology, inflammatory-stimulation electrophysiology, and cell-damage and survival assays. For molecular and histological characterization, cartilage samples were obtained from three different rats: one for RT-PCR, one for Western blotting, and one for immunofluorescence. Each assay therefore involved one animal, without independent biological replication within assays. These three animals were already included in the other experimental groups and did not increase the total number of animals used.

Animals were euthanized by carbon dioxide inhalation. Rats were placed in a euthanasia chamber, and 100% CO_2_ was introduced at a displacement rate of 30–70% of the chamber volume per minute. Following CO_2_ exposure, death was confirmed by the absence of a heartbeat. The absence of a withdrawal response to paw pinch was also verified before articular cartilage was collected under sterile conditions. Tissue intended for RNA or protein extraction was either processed immediately or frozen in liquid nitrogen. Samples used for immunofluorescence were fixed as described below, whereas cartilage intended for primary chondrocyte isolation was subjected to enzymatic digestion immediately after collection.

### 4.2. Isolation and Primary Culture of Articular Chondrocytes

Primary articular chondrocytes were isolated as previously described [9,12]. Minced articular cartilage was incubated with 0.2% type II collagenase in PBS for 2–3 h at 37 °C. Following enzymatic digestion, cells were mechanically dissociated by gentle repeated pipetting, washed and centrifuged twice, and resuspended in DMEM supplemented with 10% calf serum and penicillin (100 U/mL)-streptomycin (100 µg/mL) (In Vitro, Cat. A-01, Roma Sur, Mexico). The culture medium was replaced every two days.

For electrophysiological experiments, freshly isolated chondrocytes were seeded onto glass coverslips at a suspension density of 1–3 × 10^5^ cells/mL and maintained at 37 °C in a humidified atmosphere containing 5% CO_2_. Cells were studied shortly after isolation (day 0) or after 1, 3, or 5 days in primary culture, depending on the experimental protocol. For the culture-time experiments described below, cells were plated at approximately 500 cells/mm^2^.

For metabolic-activity and fluorescence-imaging experiments, chondrocytes were seeded in multiwell culture plates at the densities specified for each assay.

### 4.3. RT-PCR Analysis

Articular cartilage samples were immediately frozen in liquid nitrogen and subsequently ground. Total RNA was extracted using TRIzol reagent (Invitrogen, Waltham, MA, USA), and RNA concentration was determined by UV spectrophotometry. Complementary DNA was synthesized using SuperScript III reverse transcriptase and oligo(dT)_12–18_ primers (Invitrogen).

PCR amplification was performed using a PCR System 2400 thermal cycler (Perkin Elmer, Waltham, MA, USA). Two independent primer pairs targeting rat Kcna3/Kv1.3 (RefSeq NM_019270.4) were designed using NCBI Primer-BLAST (https://www.ncbi.nlm.nih.gov/tools/primer-blast/, accessed on 1 October 2023) [41]. Primer sequences, nucleotide positions, and expected product sizes are summarized in Table 1.

The amplification protocol consisted of an initial denaturation at 94 °C for 3 min, followed by 35 cycles of denaturation at 94 °C for 20 s, annealing at 60 °C for 15 s, and extension at 72 °C for 30 s. A final extension was performed at 72 °C for 10 min, after which samples were held at 4 °C. Amplification products were separated by agarose gel electrophoresis and visualized following ethidium bromide staining.

### 4.4. Western Blot Analysis

Articular cartilage samples were minced and immediately frozen in liquid nitrogen. Frozen tissue was ground using a mortar and pestle and lysed for 15 min at 4 °C in RIPA buffer supplemented with PMSF, sodium orthovanadate, and a protease-inhibitor cocktail (Cat. sc-24948A, Santa Cruz Biotechnology, Dallas, TX, USA). Lysates were transferred to 1.5-mL microcentrifuge tubes, homogenized using an ultrasonic processor (Model GEX 130), and centrifuged at 14,000 rpm for 20 min at 4 °C.

Supernatants were collected, and protein concentrations were determined using a BCA Protein Assay (Cats. 23228 and 23224, Thermo Fisher Scientific, Waltham, MA, USA). Samples were mixed with 4× loading buffer and heated at 90 °C for 5 min. Proteins (50 µg per lane) were separated by SDS-PAGE using 8% gels at 100 V for 90 min and transferred to PVDF membranes (Cat. RPN303F, Amersham Life Science, Slough, UK) using a semi-dry transfer system (Cat. 1703940, Bio-Rad, Hercules, CA, USA) at 35 V for 30 min.

Membranes were blocked for 1 h at room temperature in 5% semi-skimmed milk and 3% bovine serum albumin and incubated overnight at 4 °C with rabbit anti-Kv1.3 antibody (1:250; Cat. APC-002, Alomone Labs, Jerusalem, Israel) and mouse anti-actin antibody (1:1000; provided by Dr. Manuel Hernandez, CINVESTAV, Mexico City, Mexico). Membranes were washed with TBS-T containing 0.01% Tween 20 and incubated for 45 min at room temperature with HRP-conjugated anti-rabbit or anti-mouse secondary antibodies (Cats. 656120 and 626520, respectively; Thermo Fisher Scientific, Waltham, MA, USA). Immunoreactive bands were detected using Immobilon Western reagent (Cat. WBKLS0500, Millipore, Burlington, MA, USA), and images were acquired using a FUSION FX6-XT imaging system (WITEC AG, Sursee, Switzerland).

### 4.5. Immunofluorescence Analysis of Articular Cartilage

Articular cartilage specimens were fixed by immersion in 4% paraformaldehyde in PBS for 48 h at 4 °C and subsequently cryoprotected in 12% sucrose in Dulbecco’s phosphate-buffered saline (pH 7.3) for 24 h. Sections 20 µm thick were prepared using a cryostat (MC1510, Leica Microsystems GmbH, Wetzlar, Germany) and stored in cryopreservation solution containing 4% ethylene glycol and 1% polyvinylpyrrolidone in 0.1 M PBS, pH 7.3.

Sections were permeabilized for 1 h in PBS containing 0.2% Triton X-100, washed three times, and blocked for 2 h at room temperature in PBS containing 0.05% Triton X-100 and 4% BSA. Sections were incubated overnight at 4 °C with anti-Kv1.3 antibody (Cat. APC-002, Alomone Labs) diluted 1:200 in PBS containing 0.05% Triton X-100, 4% BSA, and 0.1% sodium azide. After washing, sections were incubated for 1 h with biotin-conjugated goat anti-rabbit IgG (ZYMED, San Francisco, CA, USA; 1:200), followed by FITC-conjugated streptavidin (ZYMED; 1:50) for 1 h.

Sections were subsequently counterstained with propidium iodide, washed, mounted with Vectashield mounting medium (Vector Laboratories, Newark, CA, USA), covered with coverslips, and sealed. Fluorescence images were acquired using a Leica TCS SP8 confocal microscope (Leica Microsystems CMS GmbH, Mannheim, Germany).

### 4.6. Electrophysiological Recordings

#### 4.6.1. Whole-Cell Patch-Clamp Recordings

Whole-cell recordings were performed according to previously established procedures [9,12]. Coverslips containing primary chondrocytes were transferred to a recording chamber mounted on an inverted microscope (Diaphot 300, Nikon, Japan) and continuously superfused with extracellular recording solution. Recordings were performed at room temperature.

An electrical connection between the bath and the reference electrode was established using U-shaped glass tubing containing 2% agarose prepared in 500 mmol/L KCl. The reference electrode was placed in a reservoir containing 500 mmol/L KCl. Patch pipettes were fabricated from borosilicate glass tubing (Cat. No. 34500-99, Kimble Chase, Vineland, NJ, USA) using a horizontal puller (P-87, Sutter Instrument Co., Novato, CA, USA) and had resistances of 2–5 MΩ when filled with intracellular solution. Pipettes were positioned using a piezoelectric-driven micromanipulator (PCS6000, Burleigh Co., Bismarck, ND, USA).

After formation of a high-resistance seal (≥2 GΩ, typically approximately 5 GΩ), the whole-cell configuration was established by applying gentle suction. Membrane currents were recorded using a patch-clamp amplifier (8900, DAGAN Corp., Minneapolis, MN, USA) controlled with pClamp 8.0 software (Axon Instruments Inc., Grand Terrace, CA, USA). Current signals were low-pass filtered at 10 kHz. Series resistance was compensated by 50–70%, and membrane potentials were corrected for the liquid junction potential.

#### 4.6.2. Voltage-Clamp Protocol and Pharmacological Isolation of Kv1.3-Sensitive Currents

Voltage-dependent K^+^ currents were elicited from a holding potential of −80 mV by applying 500-ms voltage steps from −80 to +80 mV in 10-mV increments. Whole-cell currents were recorded first under control conditions and subsequently during application of either ShK-186 (150 pM) or PAP-1 (5 nM).

The Kv1.3-sensitive current was operationally defined as the component inhibited by the corresponding blocker and was obtained by digital subtraction:

*I_Kv_*_1.3_ = *I_control_* − *I_blocker_*

Peak current measured during each voltage step was normalized to membrane capacitance to obtain current density. Conductance was calculated from the peak current according to:

$$G=\frac{I}{V_{m}-E_{K}}$$

where Vm is the test potential and E_K_ is the K^+^ equilibrium potential. An E_K_ value of −79 mV was used for conductance calculations. Conductance density was obtained by normalization to membrane capacitance.

For analysis of voltage-dependent activation, conductance values were normalized to the maximum conductance (G/Gmax) and fitted with a Boltzmann function.

$$\frac{G(V)}{\mathrm{Gmax}}=\frac{1}{1+e^{\left(\frac{V_{1/2}-V}{k}\right)}}$$

where V is the membrane potential, V_1/2_ is the half-activation voltage and k is the slope factor. Data were fitted by nonlinear regression using SigmaPlot 14.5 software.

For the initial pharmacological characterization, voltage-clamp recordings were obtained from four rats per blocker, using separate sets of animals for ShK-186 and PAP-1. These animals were subsets of the corresponding five-rat sets used for current-clamp experiments. Five cells were analyzed per rat. For each variable and voltage, cell-level measurements were averaged within each rat, and mean values and SEM were calculated across the four rat-level means.

#### 4.6.3. Current-Clamp Measurements of Membrane Potential and Input Resistance

The effects of Kv1.3 inhibition on resting membrane potential (Vm) and membrane input resistance (Rin) were evaluated under whole-cell current-clamp conditions. Vm was continuously monitored before, during, and after application of ShK-186 (150 pM) or PAP-1 (5 nM). Repeated hyperpolarizing current pulses of constant amplitude were applied throughout each recording to monitor Rin. Input resistance was calculated from the voltage deflection induced by the current pulse according to:

Rin = ∆V/∆I

Blocker-induced changes were calculated as:

∆Vm = Vm(blocker) − Vm(control)

and

∆Rin = Rin(blocker) − Rin(control)

Current-clamp recordings were obtained from five rats per blocker, using separate sets of animals for ShK-186 and PAP-1 (10 rats in total). Five cells were analyzed per rat. Cell-level measurements were averaged within each rat to obtain one biological-replicate value.

#### 4.6.4. Membrane Capacitance and Culture-Dependent Electrophysiological Measurements

Membrane capacitance was estimated from capacitive transients evoked by a −10-mV hyperpolarizing voltage step. Capacitive currents were recorded at a sampling frequency of 10 kHz, and membrane capacitance was calculated offline by integrating the area under the capacitive transient and dividing the integrated charge by the voltage-step amplitude. The analysis was performed using the Clampfit module of pClamp 8.0 (Molecular Devices, San Jose, CA, USA). Where required, estimated membrane area was calculated assuming a specific membrane capacitance of 1 µF/cm^2^ [42].

To examine culture-dependent changes in electrophysiological properties, four independent primary chondrocyte preparations were studied on days 0, 1, 3, and 5 after plating. Each preparation was generated by pooling cells isolated from the hip articular cartilage of three rats. Five cells per preparation were recorded at each time point. Total K^+^ conductance and the PAP-1-sensitive component attributed to Kv1.3 were determined using the voltage-clamp protocol described above. Measurements were averaged within each preparation and time point to obtain one independent value per preparation for statistical analysis.

### 4.7. Pro-Inflammatory Treatments

For inflammatory-stimulation electrophysiology experiments, primary rat articular chondrocytes were maintained in culture for 24 h and subsequently exposed for 12 h to IL-1α (10 ng/mL), IL-1β (10 ng/mL), or LPS (1 µg/mL). Matched untreated cultures were maintained in parallel. Following treatment, whole-cell K^+^ currents were recorded before and during application of PAP-1 (5 nM). The PAP-1-sensitive component was obtained by digital subtraction and was operationally attributed to Kv1.3.

Four independent biological replicates were performed for each inflammatory agent, each using chondrocytes isolated from the hip articular cartilage of a different rat. Separate sets of four rats were used for IL-1α, IL-1β, and LPS, yielding 12 rats in total. Within each preparation, coverslips were distributed between matched untreated and inflammatory-treatment conditions. Five cells were recorded per rat and condition, yielding 20 cell recordings per condition for each inflammatory agent. Measurements were averaged within each rat and condition, and the resulting rat-level means were used for paired statistical analysis.

For metabolic-activity, PI-positivity, and cell-density assays, primary chondrocytes were exposed for 12 h to IL-1α (10 ng/mL) or IL-1β (10 ng/mL), either alone or in combination with ShK-186 (150 pM) or PAP-1 (5 nM). Untreated cultures and cultures exposed to either blocker alone were included as controls. Four independent biological experiments were performed, each using a pooled primary chondrocyte preparation obtained from the hip articular cartilage of three rats.

### 4.8. Assessment of Chondrocyte Damage and Survival

#### 4.8.1. MTT Assay of Cellular Metabolic Activity

Cellular metabolic activity was assessed using the MTT [3-(4,5-dimethylthiazol-2-yl)-2,5-diphenyltetrazolium bromide] reduction assay. Primary chondrocytes were seeded into 96-well plates at 10,000 cells per well and allowed to attach for 12 h before experimental treatment. Cells were then exposed to the indicated pro-inflammatory cytokines and/or Kv1.3 blockers for 12 h.

MTT was added to a final concentration of 0.5 mg/mL, and cultures were incubated for 3 h at 37 °C. The medium was then removed, and the resulting formazan crystals were dissolved in dimethyl sulfoxide (DMSO). Absorbance was measured at 570 nm using a Model 680 Microplate Reader (Bio-Rad Laboratories, Hercules, CA, USA). Normalized MTT reduction was used as an index of cellular metabolic activity at the culture level.

Four independent biological experiments were performed, each using a pooled primary chondrocyte preparation obtained from three rats. Five technical replicate wells were analyzed per condition within each biological experiment. Background-corrected absorbance values were first averaged across the five wells within each biological preparation and treatment condition. The resulting preparation-level means were divided by a common reference value, calculated as the mean background-corrected absorbance of untreated control cultures from multiple biological preparations, and multiplied by 100. The four normalized preparation-level means per condition were used as the independent observations for statistical analysis.

#### 4.8.2. Propidium Iodide-Based Assessment of Cell Death

Primary chondrocytes were seeded in 96-well plates at 10,000 cells per well and allowed to attach for 12 h before experimental treatment. Loss of plasma-membrane integrity was assessed by determining the fraction of propidium iodide (PI)-positive cells. Following experimental treatment, cultures were incubated with Hoechst 33342 (4 µg/mL; Cat. No. 62249, Thermo Scientific, Rockford, IL, USA) and PI (10 µg/mL) for 15 min at 37 °C protected from light. Hoechst 33342 was used to identify the total nuclear population, whereas PI identified cells with compromised plasma-membrane integrity.

Fluorescence images were acquired using an Axiovert 200 M inverted microscope (Carl Zeiss, Göttingen, Germany) equipped with a 20× objective, an AxioCam MRm camera, and AxioVision 4.8 software. Images from all experimental groups were acquired using identical magnification, illumination, and exposure settings. For each treatment condition, five technical replicate wells were analyzed, and five microscopic fields were acquired from each well. Hoechst-positive and PI-positive nuclei were quantified using Fiji (ImageJ 1.54g) image-analysis software [43]. Identical image-processing and thresholding criteria were applied to all experimental groups.

The percentage of PI-positive cells was calculated as:

$$\text{PI-positive}\;\text{cells}\;\left(\%\right)=100\times \frac{N_{\mathrm{PI}+}}{N_{H+}}$$

where N_PI+_ represents the number of PI-positive nuclei and N_H+_ represents the total number of Hoechst-positive nuclei within the same microscopic field.

Four independent biological experiments were performed. The five fields acquired from each well were averaged to obtain one well-level value; the five well-level values were subsequently averaged to obtain one value per condition for each biological experiment. Biological-experiment means were used as the independent values for statistical analysis.

#### 4.8.3. Cell-Density Measurements

Total cell density was determined from the same Hoechst 33342 fluorescence images acquired for the cell-death assay. Hoechst-positive nuclei were quantified using Fiji (ImageJ) [43], and the number of nuclei within each microscopic field was normalized to the calibrated field area. Cell density was expressed as nuclei/mm^2^.

Images from all experimental conditions were acquired using the same 20× objective, field dimensions, and acquisition settings. For each treatment condition, five technical replicate wells were analyzed, with five microscopic fields acquired from each well. Measurements from the five fields within each well were averaged to obtain one well-level value, and the five well-level values were subsequently averaged within each biological experiment and condition. Four independent biological experiments were performed, and the resulting biological-replicate means were used as independent values for statistical analysis.

### 4.9. Recording Solutions and Pharmacological Reagents

The extracellular recording solution contained (in mM): 110 NaCl, 5 KCl, 1 MgCl_2_, 1.8 CaCl_2_, and 10 HEPES, adjusted to pH 7.4. The intracellular pipette solution contained (in mM): 5 sodium gluconate, 115 potassium gluconate, 0.1 CaCl_2_, 1 MgCl_2_, and 10 HEPES, adjusted to pH 7.2. Solution osmolarity and pH were adjusted as previously described [9].

PAP-1 [5-(4-phenoxybutoxy)psoralen; Cat. No. P6124, Sigma-Aldrich, Merck KGaA, Darmstadt, Germany] was used at a final concentration of 5 nM. ShK-186 (Cat. No. STS-390, Alomone Labs, Jerusalem, Israel) was used at a final concentration of 150 pM. Recombinant rat IL-1α (Cat. No. I3901) and IL-1β (Cat. No. I2393), as well as lipopolysaccharide (LPS) from Escherichia coli O111:B4 (Cat. No. L2630), were obtained from Sigma-Aldrich (Merck KGaA, Darmstadt, Germany). IL-1α and IL-1β were used at 10 ng/mL, whereas LPS was used at 1 µg/mL.

### 4.10. Data Analysis

Electrophysiological data, including peak ionic currents and membrane-capacitance measurements, were analyzed using Clampfit from pClamp 8.0 (Molecular Devices). Current density was calculated by dividing peak whole-cell current by membrane capacitance. Conductance density was calculated from current density using the electrochemical driving force for K^+^, with E_K_ set to −79 mV.

PAP-1- or ShK-186-sensitive currents were obtained by digital subtraction of currents recorded in the presence of blocker from the corresponding control currents. For voltage-dependence analyses, conductance was normalized to maximal conductance, and activation curves were fitted with a Boltzmann function.

For MTT measurements, absorbance values were corrected for background and normalized to untreated controls. For fluorescence-based assays, PI-positive cells were expressed as a percentage of the total nuclear population, and total cell density was expressed as nuclei/mm^2^. Technical measurements obtained within the same biological replicate were averaged before inferential statistical analysis.

### 4.11. Statistical Analysis

Statistical analyses were performed using SigmaPlot for Windows version 15 and Microsoft Excel 365. Python 3.12.14 with SciPy 1.18.1 was used for the culture-time and cell-damage analyses. Data are expressed as mean ± SEM unless otherwise stated. The independent experimental unit was the rat or independent primary chondrocyte preparation, according to the experimental design. Measurements from individual cells, technical replicate wells, or microscopic fields were averaged within the corresponding biological replicate before inferential statistical analysis.

For current-clamp experiments, blocker-induced changes in membrane potential and input resistance were calculated within each cell and averaged across the five cells recorded from each rat. Normality of rat-level changes was assessed using the Shapiro–Wilk test in SigmaPlot, with no statistically significant departures from normality detected for either outcome with either blocker (all *p* > 0.05). The resulting rat-level changes were analyzed using two-tailed one-sample Student’s *t*-tests against zero (n = 5 rats per blocker).

For culture-time experiments, each independent biological replicate consisted of a primary chondrocyte preparation generated by pooling cells from three rats (n = 4 preparations). Measurements from five cells per preparation and time point were averaged. The relative PAP-1-sensitive contribution was calculated for each cell before averaging within each preparation. Culture-dependent changes were analyzed using one-way repeated-measures ANOVA with Greenhouse–Geisser correction, with preparation as the matched unit across days 0, 1, 3, and 5. Normality was assessed using the Shapiro–Wilk test for ANOVA residuals and for the within-preparation differences between each subsequent time point and day 0. No statistically significant departures from normality were detected for the paired differences in total or PAP-1-sensitive maximal conductance density (all *p* > 0.05). For these two outcomes, days 1, 3, and 5 were compared with day 0 using two-tailed paired Student’s *t*-tests, with Holm adjustment across the three comparisons for each outcome.

For inflammatory-stimulation electrophysiology experiments, four independent rats were used for each agent. Within each rat, measurements from five cells per condition were averaged to obtain matched control and treatment means. Total conductance density, PAP-1-sensitive conductance density, and the relative PAP-1-sensitive contribution were compared using two-tailed paired Student’s *t*-tests on the four matched pairs of rat-level means, with differences defined as control minus treatment. Normality of the paired differences was assessed using the Shapiro–Wilk test. For the relative PAP-1-sensitive contribution following IL-1α exposure, the paired differences departed significantly from normality; therefore, an exact two-sided Wilcoxon signed-rank test was additionally performed as a sensitivity analysis. Both results were reported, and this comparison was interpreted cautiously. *p* values for the inflammatory-stimulation electrophysiology comparisons were not adjusted for multiple comparisons.

For MTT absorbance, PI positivity, and cell density, five technical replicate wells per condition were averaged within each of four independent biological preparations. Data were analyzed using two-way repeated-measures ANOVA, with inflammatory treatment (untreated, IL-1α, or IL-1β) and blockade (none, ShK-186, or PAP-1) as within-preparation factors. Initial ANOVA diagnostics in SigmaPlot included Shapiro–Wilk normality and Brown–Forsythe equal-variance tests. Shapiro–Wilk testing did not detect statistically significant departures from normality for MTT, PI positivity, or cell density (*p* = 0.965, 0.959, and 0.497, respectively). Brown–Forsythe testing indicated unequal variances for MTT (*p* < 0.05), but not for PI positivity or cell density. Greenhouse–Geisser correction was applied to the degrees of freedom for both main effects and their interaction to account for potential deviations from sphericity. Given the evidence of unequal variances, the MTT ANOVA results were interpreted cautiously.

Specific treatment differences in the cell-damage assays were evaluated using two-tailed paired Student’s *t*-tests on preparation-level means, with variability estimated separately from the four within-preparation differences for each contrast. Holm–Šidák adjustment was applied separately to each family of three pairwise comparisons: blockade levels within each inflammatory condition and inflammatory conditions within each blockade level. Normality of paired differences was assessed using the Shapiro–Wilk test. For reported contrasts showing evidence of non-normal differences, unadjusted exact two-sided Wilcoxon signed-rank tests were performed as sensitivity analyses, and discrepancies between approaches were reported.

Given the small biological sample sizes, nonsignificant results from normality tests were interpreted cautiously and were not considered proof of normality. Two-sided 95% confidence intervals for mean within-rat or within-preparation differences were calculated using Student’s t distribution and were not adjusted for multiple comparisons. Statistical significance was defined as *p* < 0.05, using multiplicity-adjusted *p* values where applicable.

### 4.12. Experimental Design and Measures to Minimize Bias

For electrophysiological and cell-damage experiments, sample sizes of four to five independent biological replicates were selected based on investigator judgment, without a formal prospective statistical calculation. Separate sets of five rats were used for ShK-186 and PAP-1, yielding 10 rats in total for the initial electrophysiological experiments. Within each set, four rats contributed to voltage-clamp experiments and all five contributed to current-clamp measurements. The use of five rats per blocker for current-clamp experiments was based on experimental feasibility. No study-specific written protocol was prepared before data collection. The study was not preregistered. No primary outcome measure was designated before data collection. Independent biological replicates consisted of individual rats or independently generated primary chondrocyte preparations, as specified for each experiment. For pooled-culture experiments, each preparation combined cells from three rats and constituted one biological replicate. Measurements from individual cells, technical replicate wells, and microscopic fields were averaged within the corresponding biological replicate before inferential statistical analysis. For inflammatory-stimulation and cell-damage experiments, untreated and treated cultures were generated from the same biological preparation and analyzed as matched observations. Cells with seal resistance <2 GΩ were excluded from electrophysiological recordings according to a criterion established before data collection. The number of cells excluded on this basis could not be recovered and remains unavailable. Sample sizes for each experiment are reported in the corresponding methods and figure legends. Rats and coverslips were allocated to experimental conditions without a deliberate ordering scheme; no formal randomization procedure was used. Investigators were aware of treatment identity during experimental procedures, outcome assessment, and data analysis; no blinding was implemented. Identical image-acquisition settings and image-processing criteria were applied across treatment groups. No specific strategy was used to control potential confounding related to well or coverslip position or to the order of treatment application and outcome measurements across experimental conditions.

## 5. Conclusions

The present study provides molecular and pharmacological evidence supporting a functional contribution of Kv1.3 to the electrical properties of rat articular chondrocytes. PAP-1 and ShK-186 revealed blocker-sensitive outward currents, and blockade with either compound depolarized the membrane and increased input resistance. During primary culture, total and PAP-1-sensitive conductance densities decreased, while no statistically significant change in the relative PAP-1-sensitive contribution was detected.

In unadjusted paired analyses, IL-1α and IL-1β increased PAP-1-sensitive conductance. Following IL-1β exposure, this increase was accompanied by reduced total conductance and an increased relative PAP-1-sensitive contribution. The relative increase observed following IL-1α requires cautious interpretation because the paired differences departed from normality and the exact Wilcoxon sensitivity analysis was nonsignificant. No statistically significant changes in the conductance endpoints were detected following LPS exposure under the conditions tested.

Pharmacological blockade also attenuated cytokine-associated changes in metabolic activity and PI positivity, with effects on cell density depending on the treatment combination. These findings support further investigation of Kv1.3-sensitive mechanisms in chondrocyte responses to inflammatory stress. Genetic validation, independent replication, and direct measurements of downstream signaling are needed to establish the mechanisms and their relevance to cartilage disease.

## Figures and Tables

**Figure 1:**
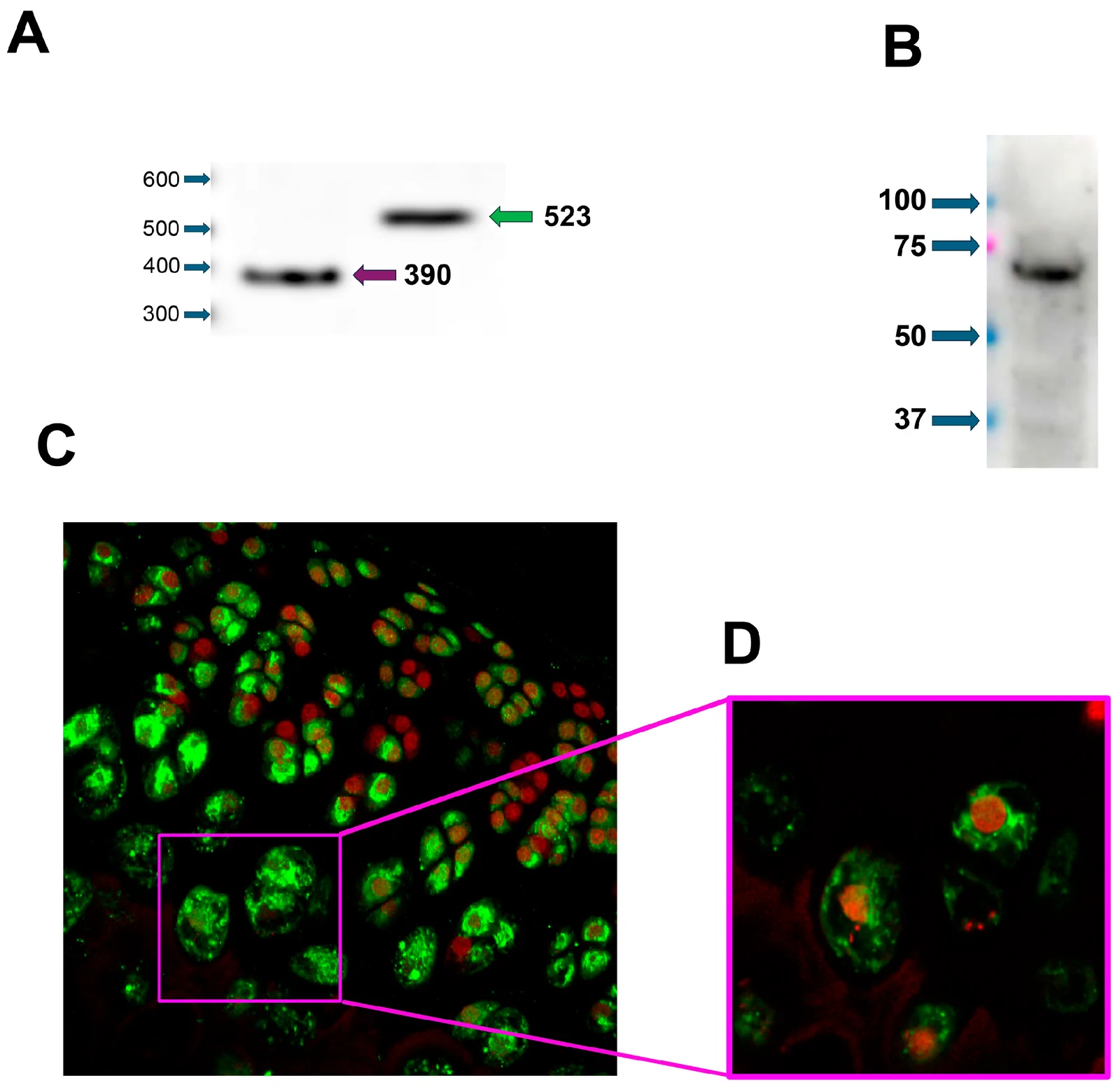
Kv1.3 expression in rat articular cartilage**.** (**A**) RT-PCR products obtained from articular cartilage. Arrows indicate bands at approximately 390 and 523 bp. (**B**) Western blot showing a prominent Kv1.3-immunoreactive band at approximately 70 kDa. (**C**) Immunofluorescence image of a rat articular cartilage section showing anti-Kv1.3 labeling in green and nuclei stained with propidium iodide in red. (**D**) Enlarged view of the boxed region in (**C**) showing Kv1.3 labeling in individual chondrocytes. RT-PCR, Western blotting, and immunofluorescence were each performed using cartilage from a different rat (n = 1 animal per assay; three animals in total), without independent biological replication within assays.

**Figure 2:**
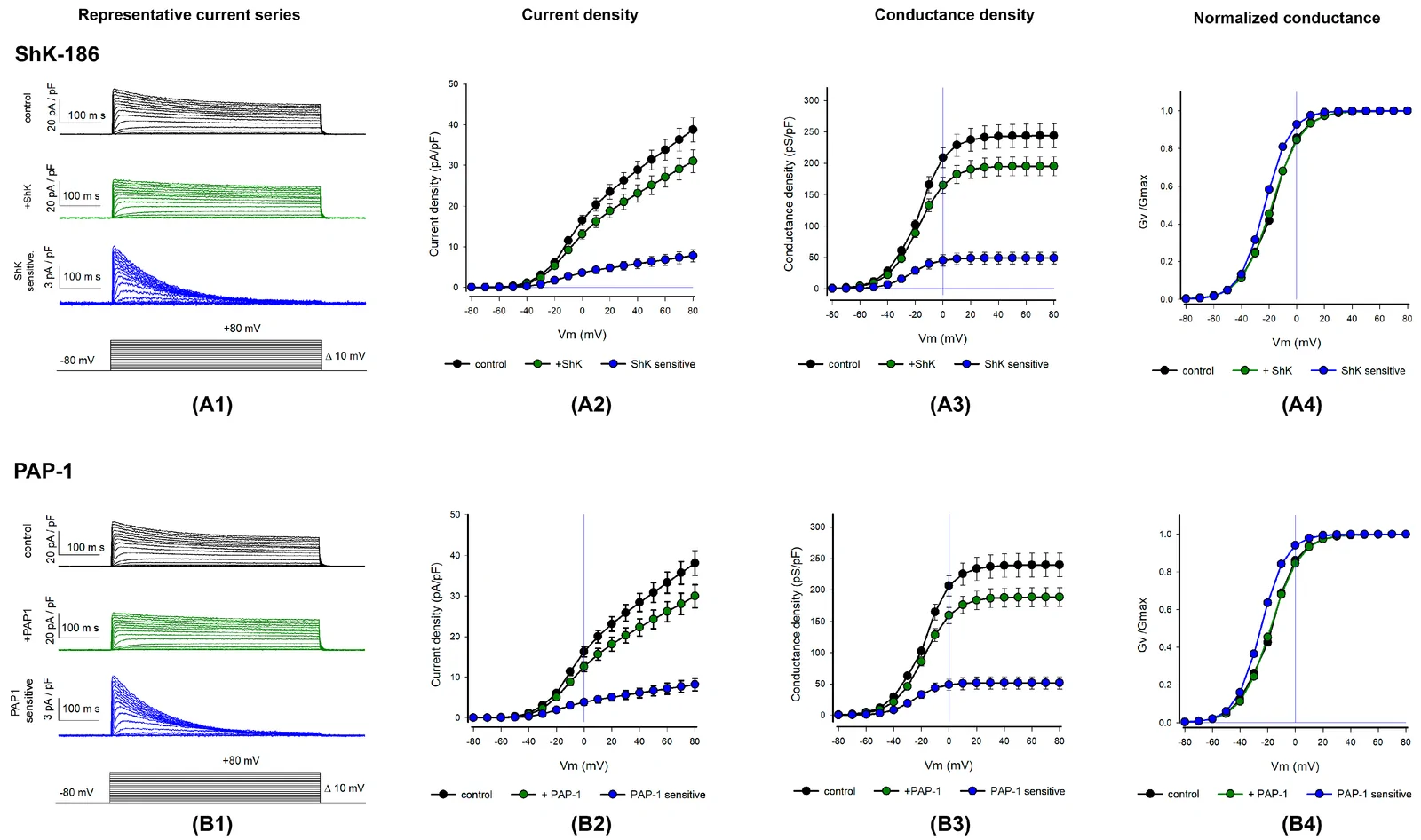
ShK-186- and PAP-1-sensitive potassium-current components in freshly isolated rat articular chondrocytes. (**A**) Effects of ShK-186 (150 pM) and (**B**) effects of PAP-1 (5 nM). (**A1**,**B1**) Representative whole-cell current families recorded under control conditions (top), in the presence of the corresponding blocker (middle), and the blocker-sensitive component obtained by digital subtraction (bottom). Currents were elicited from a holding potential of −80 mV using voltage steps from −80 to +80 mV in 10-mV increments. (**A2**,**B2**) Peak current density–voltage relationships under control conditions, during blocker application, and for the blocker-sensitive component. (**A3**,**B3**) Corresponding conductance density–voltage relationships. (**A4**,**B4**) Voltage dependence of activation expressed as normalized conductance (g/g_max_); continuous lines represent Boltzmann fits. Four independent biological replicates (rats) were analyzed for each blocker, with five cells recorded per rat (20 cells per blocker). For each variable and voltage, measurements from the five cells were averaged to obtain one rat-level value. Symbols and error bars represent the mean ± SEM calculated from the four rat-level means. Individual cells were subsamples nested within rats and were not treated as independent biological replicates.

**Figure 3:**
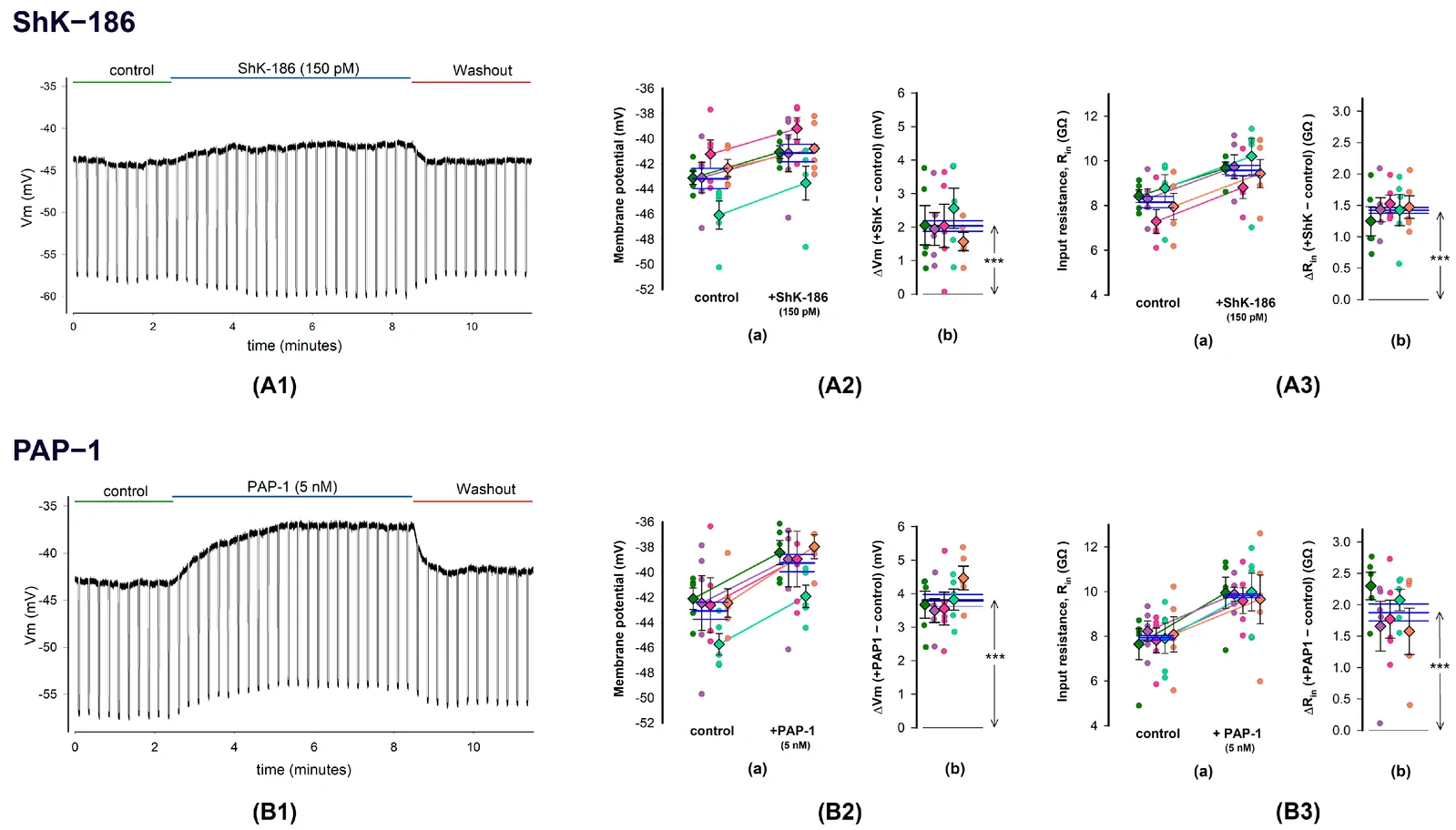
Effects of Kv1.3 blockade on resting membrane potential and input resistance in freshly isolated rat articular chondrocytes. (**A1**,**B1**) Representative membrane-potential recordings before, during, and after blocker application; repeated hyperpolarizing current pulses were used to monitor input resistance (Rin). (**A2**,**B2**) Membrane potential under control and blocker conditions (a) and blocker-induced change, ΔVm (b). (**A3**,**B3**) Input resistance under control and blocker conditions (a) and blocker-induced change, ΔRin (b). Small colored symbols represent individual cells, diamonds represent rat-level means, and blue lines indicate overall mean ± SEM. Five rats per blocker were analyzed, using separate sets of animals for ShK-186 and PAP-1 (10 rats in total), with five cells recorded per rat. Statistical significance of ΔVm and ΔRin was assessed using two-tailed one-sample Student’s *t*-tests on rat-level means versus zero. *** *p* < 0.001.

**Figure 4:**
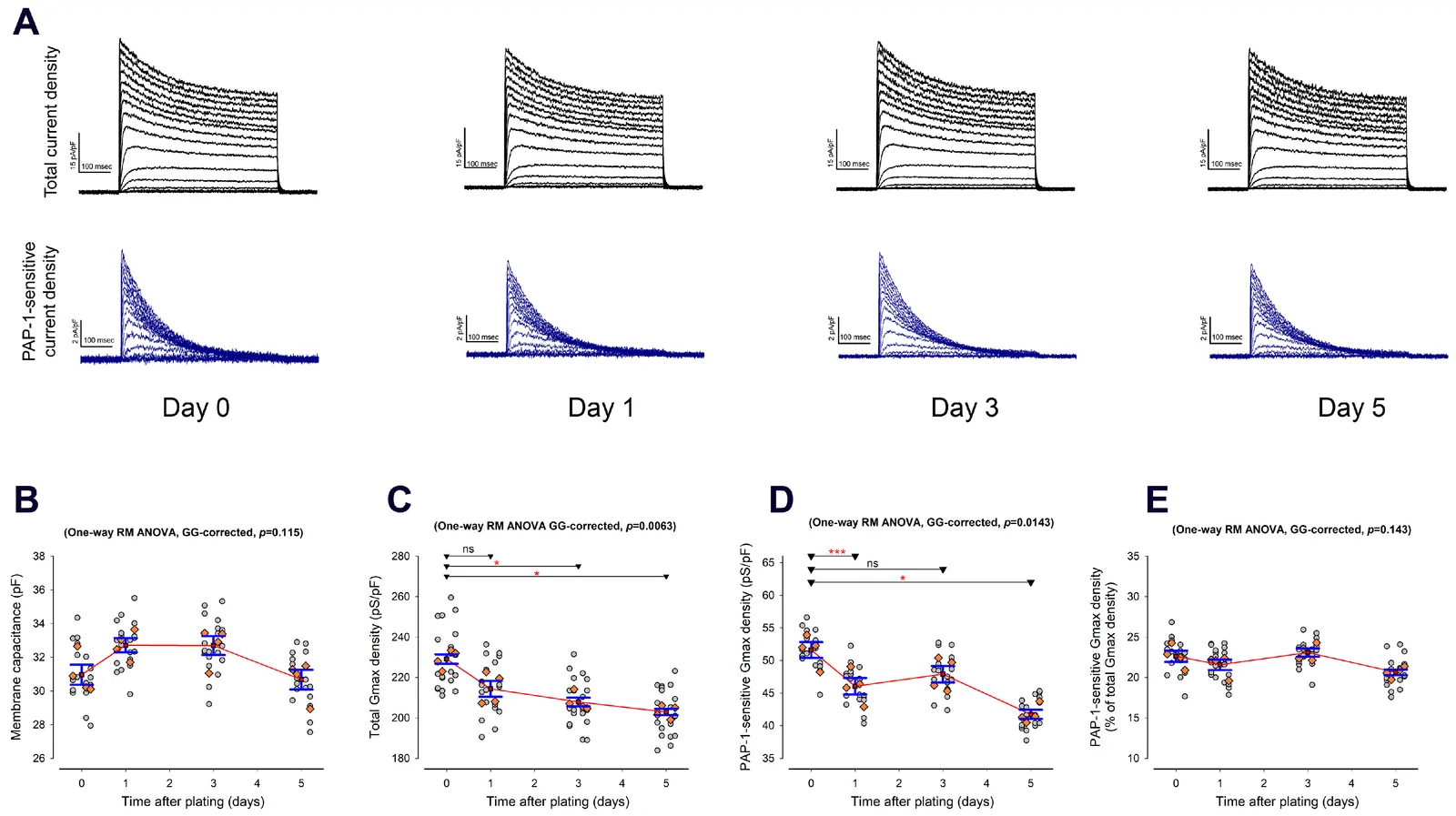
Time-dependent changes in total K^+^ conductance and the PAP-1-sensitive component attributed to Kv1.3 in primary cultured rat articular chondrocytes. (**A**) Representative current-density families on days 0, 1, 3, and 5 after plating: total (upper) and PAP-1-sensitive (lower). The latter was obtained by subtraction after PAP-1 (5 nM) application. Currents were elicited by 500-ms steps from −80 to +80 mV in 10-mV increments (holding potential, −80 mV). Scale bars: 15 pA/pF (upper), 2 pA/pF (lower), and 100 ms. (**B**) Membrane capacitance. (**C**) Total and (**D**) PAP-1-sensitive maximal conductance densities. (**E**) PAP-1-sensitive density as a percentage of total maximal conductance density, calculated per cell before averaging within preparations. Four independent preparations were analyzed, each pooling chondrocytes from three rats, with five cells per preparation and time point. Gray circles: individual cells; orange diamonds: preparation means; red squares and connecting lines: overall means; blue bars: SEM across preparations (n = 4). Analyses used preparation-level means: one-way repeated-measures ANOVA with Greenhouse–Geisser correction, with preparations matched across time (*p* values above panels), and, for C and D, two-tailed paired *t*-tests with Holm adjustment for three comparisons versus day 0 per outcome. ns, adjusted *p* ≥ 0.05; * *p* < 0.05; *** *p* < 0.001. Individual cells were subsamples, not independent biological replicates.

**Figure 5:**
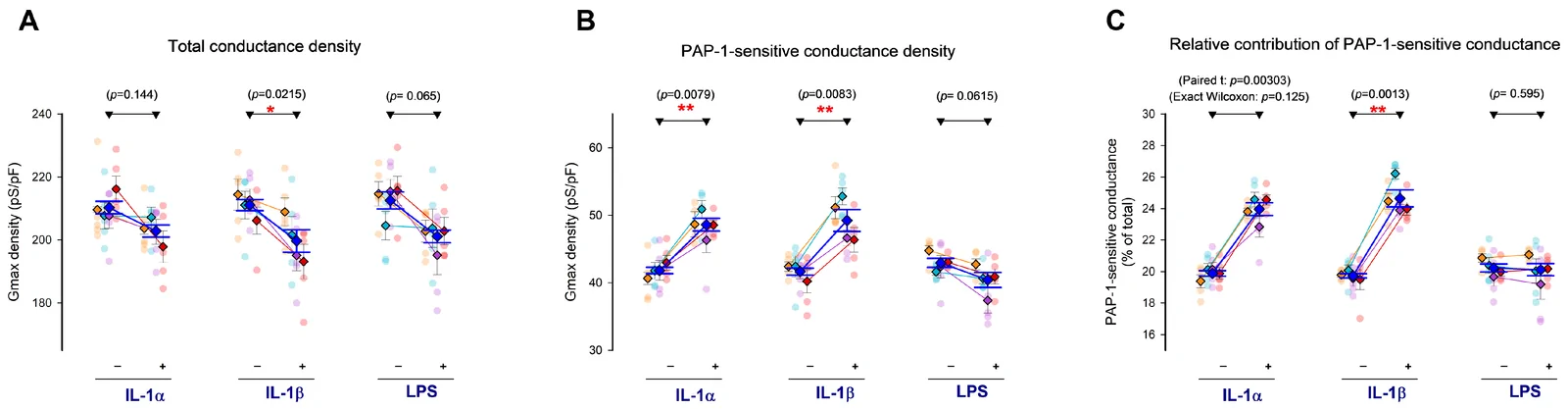
Effects of pro-inflammatory stimulation on total and PAP-1-sensitive K^+^ conductance in primary rat articular chondrocytes. Cells were cultured for 24 h and then exposed for 12 h to IL-1α (10 ng/mL), IL-1β (10 ng/mL), or LPS (1 µg/mL). Whole-cell K^+^ currents were recorded before and during PAP-1 (5 nM), and the PAP-1-sensitive component was obtained by digital subtraction and attributed to Kv1.3. (**A**) Maximal total K^+^ conductance density. (**B**) Maximal PAP-1-sensitive conductance density. (**C**) Percentage contribution of PAP-1-sensitive conductance to total K^+^ conductance. Four biological replicates were analyzed for each inflammatory agent, with five cells recorded per rat and condition. Pale symbols indicate individual cells, connected colored diamonds indicate matched rat-level means, and blue symbols with error bars indicate mean ± SEM. Comparisons were performed using two-tailed paired Student’s *t*-tests on rat-level means. For IL-1α in panel (**C**), an exact two-sided Wilcoxon signed-rank test was additionally used because paired differences failed the Shapiro–Wilk normality test. *p* values are shown above each comparison. * *p* < 0.05; ** *p* < 0.01. Individual cells were averaged within each rat and condition before analysis and were not treated as independent biological replicates. *p* values are unadjusted for multiple comparisons.

**Figure 6:**
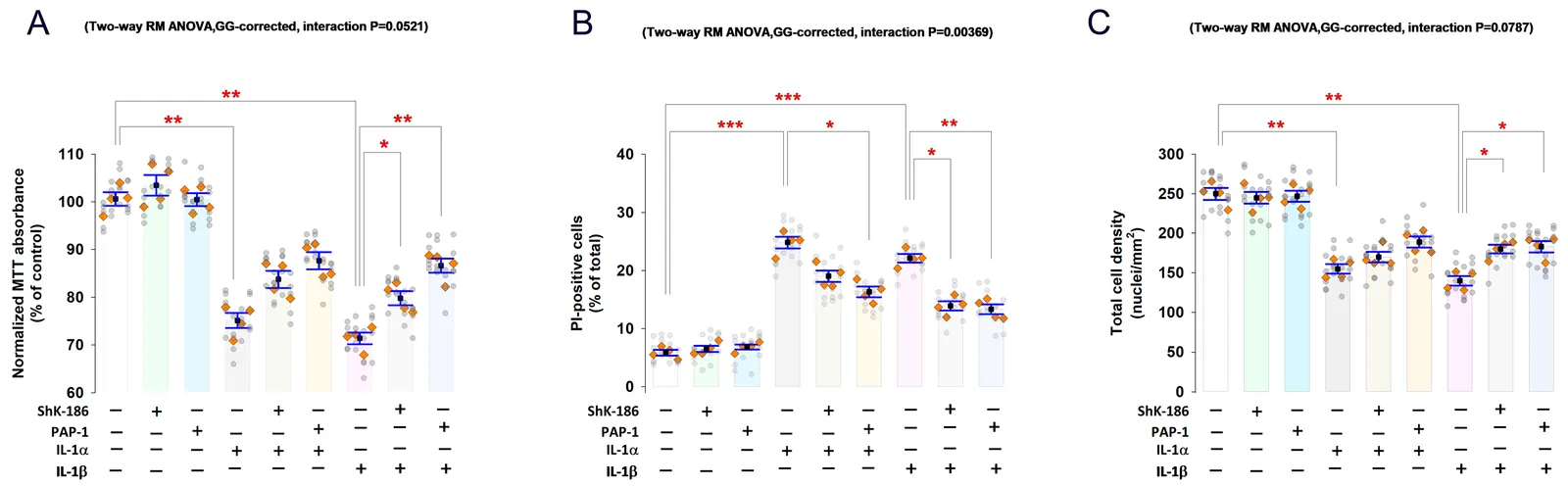
Kv1.3 blockade attenuates the detrimental effects of pro-inflammatory cytokine stimulation on rat articular chondrocytes. Primary rat articular chondrocytes were exposed for 12 h to IL-1α (10 ng/mL) or IL-1β (10 ng/mL), alone or in combination with ShK-186 (150 pM) or PAP-1 (5 nM); blocker-only conditions were also included. (**A**) MTT-formazan absorbance normalized to untreated controls. (**B**) PI-positive nuclei as a percentage of total Hoechst-positive nuclei. (**C**) Total cell density, expressed as nuclei/mm^2^. Four independent biological preparations were analyzed (n = 4), each obtained by pooling chondrocytes from three rats, with five technical replicate wells per condition and preparation. Gray dots represent individual wells; in B and C, each well-level value represents the mean of five microscopic fields. Orange diamonds represent preparation-level means, black dots indicate overall means, and blue bars indicate SEM across preparations. Data were analyzed using two-way repeated-measures ANOVA with Greenhouse–Geisser correction; *p* values above panels refer to the interaction between inflammatory treatment and blockade. Brackets indicate significant comparisons from two-tailed paired *t*-tests with Holm–Šidák adjustment within families of three pairwise comparisons among blockade levels within each inflammatory condition or among inflammatory conditions within each blockade level. * *p* < 0.05; ** *p* < 0.01; *** *p* < 0.001.

**Table 1:** Primer sequences used for RT-PCR amplification of rat *Kcna3* (Kv1.3).

RefSeq	Size (bp)	Primer	Sequence (5′–3′)	Position
NM_019270.4	390	Forward	GCAGAGGCGGTGGTGG	54-69
		Reverse	CAGCCCGGAGATGTTGATGA	443-424
	523	Forward	GACCGCTACGAACCGCTG	354-371
		Reverse	CGGGTAGTGTCTCCAAGCAG	876-857

## Data Availability

The data presented in this study are contained within the article.

## Abbreviations

The following abbreviations are used in this manuscript:

ANOVA	Analysis of variance
BSA	Bovine serum albumin
DMEM	Dulbecco’s modified Eagle’s medium
DMSO	Dimethyl sulfoxide
IL-1α	Interleukin-1α
IL-1β	Interleukin-1β
Kv1.3	Voltage-gated potassium channel 1.3
LPS	Lipopolysaccharide
MTT	3-(4,5-Dimethylthiazol-2-yl)-2,5-diphenyltetrazolium bromide
PAP-1	5-(4-Phenoxybutoxy)psoralen
PBS	Phosphate-buffered saline
PI	Propidium iodide
RT-PCR	Reverse transcription polymerase chain reaction
SEM	Standard error of the mean
